# Changes in Plasma Metabolome Profiles Following Oral Glucose Challenge among Adult Chinese

**DOI:** 10.3390/nu13051474

**Published:** 2021-04-27

**Authors:** Shaofeng Huo, Liang Sun, Geng Zong, Xia Shen, He Zheng, Qianlu Jin, Huaixing Li, Huiyong Yin, Xu Lin

**Affiliations:** 1 Shanghai Institute of Nutrition and Health, University of Chinese Academy of Sciences, Chinese Academy of Sciences, Shanghai 200031, China; sfhuo@sibs.ac.cn (S.H.); sunliang@sibs.ac.cn (L.S.); zonggeng@sibs.ac.cn (G.Z.); shenxia@shanghaitech.edu.cn (X.S.); zhenghe@sibs.ac.cn (H.Z.); qljin@sibs.ac.cn (Q.J.); lihx@sibs.ac.cn (H.L.); hyyin@sibs.ac.cn (H.Y.); 2 School of Life Science and Technology, ShanghaiTech University, Shanghai 201210, China; 3 Key Laboratory of Food Safety Risk Assessment, Ministry of Health, Beijing 100000, China; 4 Key Laboratory of Systems Health Science of Zhejiang Province, Hangzhou Institute for Advanced Study, University of Chinese Academy of Sciences, Chinese Academy of Sciences, Hangzhou 310024, China

**Keywords:** diabetes, oral glucose tolerance test, metabolome, dynamic change

## Abstract

Little is known about changes in plasma metabolome profiles during the oral glucose tolerance test (OGTT) in Chinese. We aimed to characterize plasma metabolomic profiles at 0 and 2 h of OGTT and their changes in individuals of different glycemic statuses. A total of 544 metabolites were detected at 0 and 2 h of OGTT by a nontarget strategy in subjects with normal glucose (*n* = 234), prediabetes (*n* = 281), and newly diagnosed type 2 diabetes (T2D) (*n* = 66). Regression model, mixed model, and partial least squares discrimination analysis were applied. Compared with subjects of normal glucose, T2D cases had significantly higher levels of glycerone at 0 h and 22 metabolites at 2 h of OGTT (false discovery rate (FDR) < 0.05, variable importance in projection (VIP) > 1). Seven of the twenty-two metabolites were also significantly higher in T2D than in prediabetes subjects at 2 h of OGTT (FDR < 0.05, VIP > 1). Two hours after glucose challenge, concentrations of 35 metabolites (normal: 18; prediabetes: 23; T2D: 13) significantly increased (FDR < 0.05, VIP > 1, fold change (FC) > 1.2), whereas those of 45 metabolites (normal: 36; prediabetes: 29; T2D: 18) significantly decreased (FDR < 0.05, VIP > 1, FC < 0.8). Distinct responses between cases and noncases were detected in metabolites including 4-imidazolone-5-acetate and 4-methylene-L-glutamine. More varieties of distinct metabolites across glycemic statuses were observed at 2 h of OGTT compared with fasting state. Whether the different patterns and responsiveness of certain metabolites in T2D reflect a poor resilience of specific metabolic pathways in regaining glucose homeostasis merits further study.

## 1. Introduction

An epidemic of type 2 diabetes (T2D) has affected approximately 9.3% of adults worldwide [1]. Individuals with T2D are known to have a 2-fold higher risk of cardiovascular disease (CVD), the leading cause of mortality globally [2], as well as increased risks of other noncommunicable diseases, which contribute to enormous economic and health burdens [3,4]. As a complex disease, T2D can be predicted by lifestyle, obese phenotypes, and clinical indicators. However, it was still not fully understood how disturbed metabolic pathways were linked to the progress of impaired glucose homeostasis from normal glycemia to a prediabetic condition and eventually to T2D. Thus, identifying novel metabolic signatures linked to different stages of pathophysiological disturbance and evaluating individuals’ glucose homeostatic capacity during the progression of T2D are critical for early prevention and intervention, especially for those countries with high rates of undiagnosed T2D, such as China, where 56.7% of patients were undiagnosed [5].

With recent developments in advanced metabolomics technologies, plenty of metabolic biomarkers detected in the fasting state, including branched-chain amino acids, medium- and long-chain acylcarnitines, and sphingolipids, have been found to be associated with increased T2D risk [6,7,8,9,10,11]. However, in the postprandial state, the metabolic system shifts from a catabolic to an anabolic mode. After overnight fasting, fuel is primarily supplied by glycogenolysis, lipolysis, and proteolysis, whereas after carbohydrate intake, elevated blood glucose levels will trigger insulin secretion (pancreas), suppression of glycogenolysis and gluconeogenesis (liver), lipolysis (adipose tissue), and proteolysis (muscle) to maintain fuel homeostasis [12,13,14]. Thus, fasting levels of metabolites cannot adequately represent the capability of the body to maintain homeostasis under dietary perturbations [15]. Indeed, standard nutritional challenge tests such as the “PhenFlex test” have been developed to evaluate organ and tissue functions to counteract stress and regain metabolic hemostasis. Biomarkers identified in the challenge test were shown to be more sensitive than those detected in the fasting state to discriminate diabetic cases from healthy controls [16,17]. Therefore, the determination of dynamic changes in metabolites during nutritional challenge might aid in identifying metabolites from pathways and mechanisms that are inactive during the fasting condition.

The oral glucose tolerance test (OGTT) is considered the gold standard to assess an individual’s ability to maintain and regain glucose homeostasis. The test involves administering 75 g of glucose following overnight fasting. This sudden glucose challenge induces a transition from fasting to feeding state, as well as causing a switch from catabolism to anabolism in the body [12]. Thus, OGTT provides a good opportunity to investigate dynamic changes in metabolites and metabolic pathways linked to the resilience of glucose homeostasis. To date, only a few studies have examined metabolite variations during OGTT. Their results showed that levels of some metabolites, including free fatty acids [13], amino acids [12], acylcarnitines [14], glycerol [12], β-hydroxybutyric acid [12], and hypoxanthine [12], decreased, while concentrations of others, including lactate and hippurate, increased [18]. However, most of these studies were conducted in Western nondiabetic populations with relatively small sample sizes. It is thus unclear whether their results are applicable to Chinese populations, who have different dietary patterns [19] and genetic variations involved in glucose metabolism [20]. Therefore, the current study aimed to investigate the metabolomic profiles of subjects with normal glucose, prediabetes, and T2D in a fasting state and at 2 h of OGTT, as well as the dynamic changes in the metabolome during OGTT in adult Chinese individuals of different glycemic statuses.

## 2. Materials and Methods

### 2.1. Study Population

The Guizhou-Bijie Type 2 Diabetes Study was a population-based case–control study conducted from September 2009 to January 2010 in Bijie city of Guizhou province. A total of 4917 participants aged 30 to 80 years were recruited. Participants were required to have been resident for at least 10 years in the Bijie area and to be free from the following conditions: (1) type 1 diabetes; (2) severe psychological disorders, physical disabilities, cancer, CVD, Alzheimer’s disease, or dementia within 6 months; or (3) current diagnosis of tuberculosis, AIDS, or other communicable diseases. Of the participants, 2755 underwent a standard 2 h 75 g OGTT if they had not been previously diagnosed with T2D and were not currently taking antidiabetic treatments. After excluding those with insufficient blood samples, 581 participants (234 with normal glucose, 281 with prediabetes, and 66 with newly diagnosed T2D), for whom both pre- and post-OGTT metabolomic data were available, were finally included in the current analysis. The flow chart of the current study is shown in Supplementary Figure S1. The study protocol was approved by the institutional review board of the Shanghai Institutes for Biological Sciences. Written informed consent was provided by all participants. This study abided by the principles of the Declaration of Helsinki.

### 2.2. Data Collection

Information on demographic variables, health statuses, and lifestyles was obtained by face-to-face interviews by research term members using a standardized questionnaire. Educational attainment was classified into three groups according to years of education (0–6 years, 7–9 years, or ≥10 years). Current smoking and alcohol drinking were grouped into ’yes’ or ’no’ categories. All participants were invited to undergo a physical examination in local hospitals. Anthropometric measurements were performed by trained medical staff according to a standardized protocol. Height and weight were measured in light clothing without shoes to the nearest 0.1 cm and 0.1 kg, respectively. Body mass index (BMI) was calculated as weight (kg) divided by the square of height (m). After an overnight fast, subjects underwent a 75 g OGTT with venous blood samples collected at fasting state and 2 h after OGTT.

### 2.3. Plasma Collection and Laboratory Measurements

Blood samples were collected at both 0 and 2 h of OGTT and centrifuged at 3000 rpm for 15 min (4 °C). Plasma samples were stored at −80 °C before analysis. Fasting and 2 h glucose were measured enzymatically by an automatic analyzer (7080 Hitachi, Tokyo, Japan) with reagents from Wako Pure Chemical Industries (Osaka, Japan). Fasting and 2 h insulin were measured by radioimmunoassay (LINCO Research, St. Charles, MO, USA). Homeostasis model assessment of insulin resistance (HOMA-IR) and homeostasis model assessment of β-cell function (HOMA-B) were conducted by the updated method (http://www.dtu.ox.ac.uk/homacalculator/, accessed on 30 March 2018).

### 2.4. Metabolic Profiling

All plasma samples were thawed at 4 °C on ice overnight. Quality control (QC) samples were prepared by pooling aliquots (10 μL from each sample) of all plasma samples. Analysis was performed following a procedure described previously [21]. Fifty microliter plasma samples were mixed with 150 μL cold methanol dissolved in an internal standard of inosine (15N4, 95%+) (1 ppm). Samples were vortexed for 30 s and incubated at −20 °C for 2 h for protein precipitation. After centrifugation for 20 min (4000 rpm, 4 °C), the supernatants were transferred to liquid chromatography–mass spectrometry (LC-MS) vials for ultra-high-performance LC with quadrupole time-of-flight MS (UHPLC-QTOF/MS)(UHPLC system: Nexera UHPLC LC-30A, Shimadzu Technologies, Kyoto, Japan; QTOF mass spectrometer: AB 6600 TripleTOF, SCIEX, Concord, ON, Canada) analysis. The QC samples were processed by the same method as the plasma samples. All plasma samples were randomly injected for data acquisition, whereas the blank samples (100% acetonitrile (ACN)) and QC samples were injected every 10 samples. In total, seven batches were arranged for the measurement. The LC-MS analysis was performed using a UHPLC system (Nexera UHPLC LC-30A, Shimadzu Technologies, Kyoto, Japan) coupled to a QTOF mass spectrometer (AB 6600 TripleTOF, SCIEX, Concord, ON, Canada) in positive and negative modes, respectively. The mobile phases consisted of 25 mM CH3COONH4 + 25 mM NH4OH (A) and 100% acetonitrile (B). Chromatographic separation was performed on UPLC BEH amide columns (1.7 μm, 2.1 × 100 mm) with the following gradient: 0–0.5 min, 95% B; 0.5–7 min, 95% B to 65% B; 7–8 min, 65% B to 40% B; 8–9 min, 40% B; 9–9.1 min, 40% B to 95% B; 9.1–12 min, 95% B. With R package XCMS (version 1.46) [22], the MS raw data (.wiff) files were converted to the mzXML format using ProteoWizard. The parameters in XCMS were set as follows: centwave settings for feature detection (Δm/z = 25 ppm, peak width = c (5, 30)); obiwarp settings for retention time correction (profStep = 1); and parameters including minfrac = 0.5, bw = 5, and mzwid = 0.015 for chromatogram alignment. Metabolite identification was performed using the online MetDNA server [23]. Undefined metabolites, metabolites with >20% missing, and those with a coefficient of variation >30% were excluded. Zero values were imputed using half of the minimum values in the original data. MetNormalizer was used to remove intra- and inter-batch variations [24]. Finally, 544 metabolites were analyzed.

### 2.5. Definition of Diseases

T2D was identified as fasting glucose ≥ 7.0 mmol/L or OGTT 2 h glucose ≥ 11.1 mmol/L. Among non-T2D individuals, prediabetes was defined as 5.6 ≤ fasting glucose < 7.0 mmol/L or 7.8 ≤ OGTT 2 h glucose < 11.1 mmol/L. Participants with fasting glucose < 5.6 mmol/L and OGTT 2 h glucose < 7.8 mmol/L were categorized as normal glucose subjects [25].

### 2.6. Statistical Analyses

Analysis of variance (ANOVA), Kruskal–Wallis test, and chi-squared test were used to compare baseline characteristics among normal glucose, prediabetes, and T2D subjects for normally distributed continuous variables, skewed-distribution continuous variables, and categorical variables, respectively.

A multivariate logistic regression model and partial least squares discriminant analysis (PLS-DA) were used to identify different metabolites among subjects with normal glucose, prediabetes, and T2D. PLS-DA is an algorithm that combines dimensionality reduction and discriminant analysis for predictive and descriptive modeling, as well as discriminative variable selection when dealing with high-dimensional data [26]. The logistic regression model was adjusted for age, sex, education attainment (≤6 years, 7–9 years, ≥10 years), current smoking (yes/no), current drinking (yes/no), BMI, family history of diabetes (yes/no), and measure batches. False discovery rate (FDR) was defined as expected proportion of incorrect assignments among the accepted assignments, and it was used in the current study to do multiple hypothesis testing correction to reduce the number of false positives [27]. VIP (variable importance in projection) values showed the magnitude that metabolites contributed to group separation in the PLS-DA model [28]. Metabolites with FDR < 0.05 in the logistic model and VIP > 1.0 in PLS-DA were considered as significantly different metabolites between different glycemic statuses.

Linear mixed-effects models combined with PLS-DA were employed to identify significantly changed metabolites during OGTT. The fold change (FC) of metabolites represented the geometric mean of the ratio of the metabolites in each sample at 2 h to 0 h. Significantly changed metabolites were defined by FDR-corrected *p*-value < 0.05 in the mixed-effects model, VIP > 1 in PLS-DA, and FC > 1.2 for increased metabolites or FC < 0.8 for decreased metabolites in total, normal glucose, prediabetes, or T2D subjects. Percent change was calculated as (FC − 1) × 100%.

All analyses were performed in R software (version 3.5.1, R Core Team, Vienna, Austria). Raw values of metabolites were used to calculate FC to avoid negative FC values, and log-transformed values were used in all other analyses.

## 3. Results

### 3.1. Characteristics of Participants

The characteristics of the normal glucose, prediabetes, and T2D subjects are shown in Table 1. The mean (SD) age of all participants was 48.3 (10.1) years, and 78.1% of them were men. Compared with those of normal glucose, T2D and prediabetes subjects were older and more likely to have a family history of diabetes. They also exhibited higher BMI, higher levels of fasting and 2 h glucose and insulin, and higher levels of HOMA-IR but had lower levels of HOMA-B (all *p* < 0.05).

### 3.2. Differences in Metabolites among Normal Glucose, Prediabetes, and T2D Subjects at 0 and 2 h of OGTT

**Figure 1:**
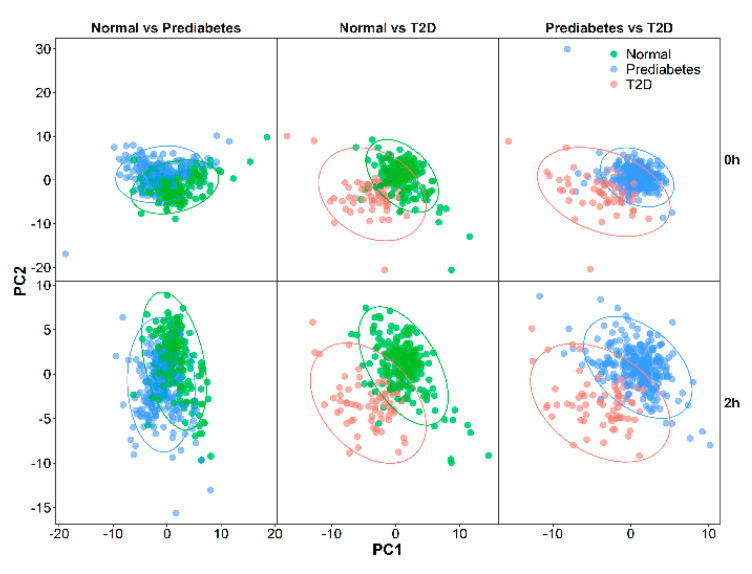
PLS-DA score plot generated from 544 metabolites at 0 and 2 h in normal glucose (*n* = 234), prediabetes (*n* = 281), and T2D (*n* = 66) subjects. T2D, type 2 diabetes; PC1, the first principal component; PC2, the second principal component.

PLS-DA score plots of the first two principal components (Figure 1) demonstrated distinct metabolomic profiles between T2D and the other two groups (normal glucose and prediabetes) at 0 and 2 h of OGTT but not between normal glucose and prediabetes subjects. After combining the results of PLS-DA and logistic regression analysis (Table 2), levels of glycerone at 0 h (odds ratio (OR) per SD 2.02 (95% CI 1.45–2.87)) and those of 22 metabolites at 2 h of OGTT (OR per SD ranging from 1.76 to 2.44) were significantly higher in T2D subjects than in normal glucose subjects. Moreover, seven of the 22 metabolites at 2 h were also significantly higher in T2D subjects than in prediabetes subjects (OR per SD ranging from 1.66 to 2.24).

### 3.3. Responses of Metabolites to the Glucose Challenge

**Figure 2:**
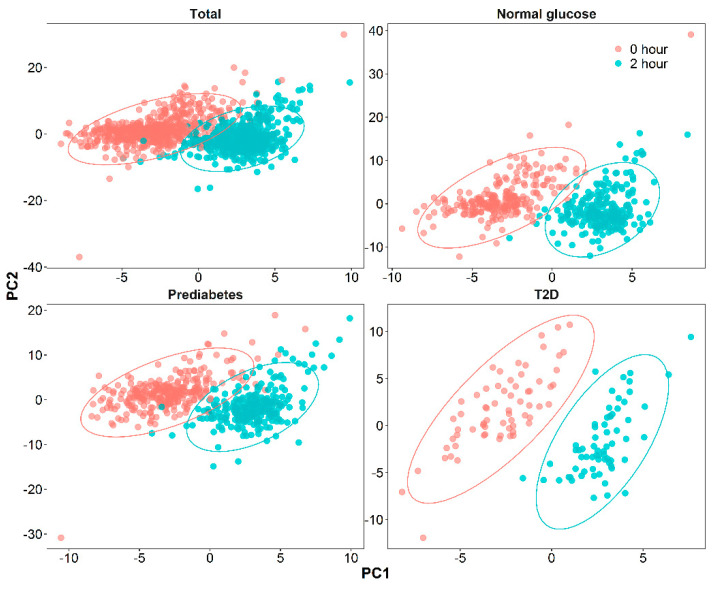
PLS-DA score plot of plasma samples drawn at 0 and 2 h of OGTT in total (*n* = 581), normal glucose (*n* = 234), prediabetes (*n* = 281), and T2D (*n* = 66) subjects.

**Figure 3:**
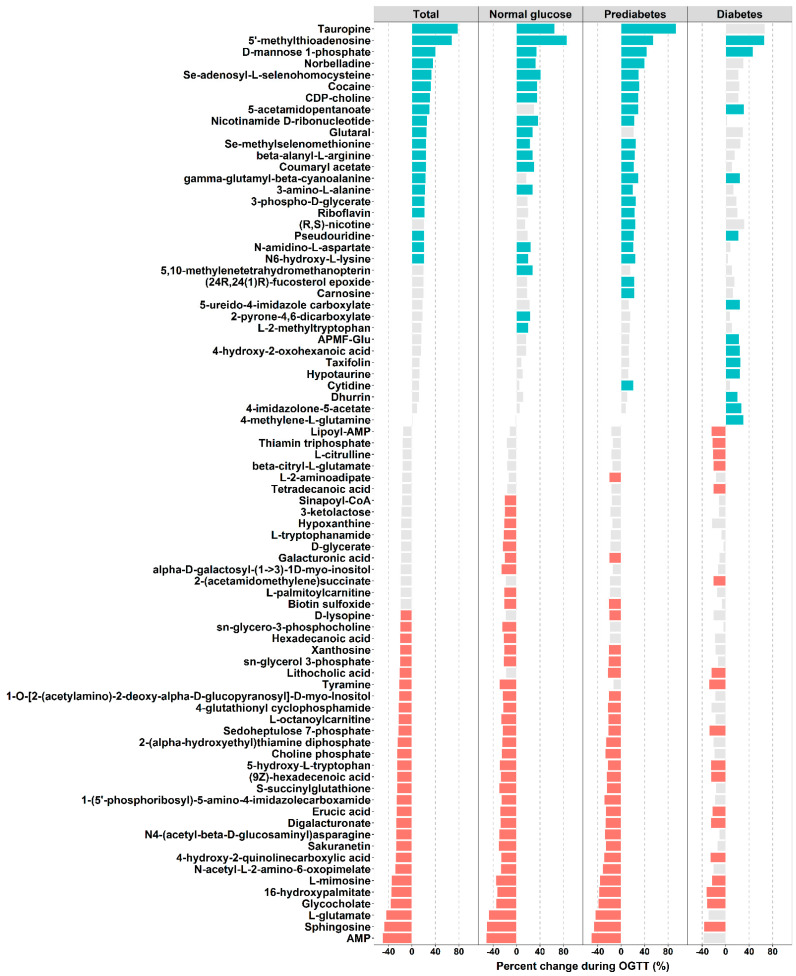
Percent changes in metabolite levels in response to oral glucose challenge. Green bars represent significantly increased metabolites. Red bars represent significantly decreased metabolites. Grey bars represent insignificantly changed metabolites. Percent change was calculated as (FC − 1) × 100%.

As presented in Figure 2, the PLS-DA score plots showed distinct metabolomic patterns at 0 and 2 h of OGTT among the various groups: all subjects and subjects with normal glucose, prediabetes, and T2D. In total, levels of 80 metabolites significantly changed during OGTT (FDR < 0.05, VIP > 1, FC > 1.2 or FC < 0.8) (Figure 3), of which levels of 35 metabolites increased (total, 20; normal glucose, 18; prediabetes, 23; T2D, 13; a Venn diagram is shown in Supplementary Figure S2) in response to OGTT, whereas those of 45 metabolites decreased (total, 29; normal glucose, 36; prediabetes, 29; T2D, 18; a Venn diagram is shown in Supplementary Figure S3). The largest increase at 2 h of OGTT was seen for tauropine, levels of which increased by 79% in all, 65% in normal, 94% in prediabetes, and 67% in T2D subjects. By contrast, the largest decrease at 2 h of OGTT was seen for levels of AMP, which decreased by 50% in all, 52% in normal, 51% in prediabetes, and 38% in T2D subjects. Notably, the patterns of significantly changed metabolites between normal glucose and prediabetes subjects were similar, whereas T2D subjects exhibited a distinct pattern (Figure 3). For example, T2D subjects, but not normal glucose and prediabetes subjects, had significantly elevated 4-imidazolone-5-acetate (amino acid metabolism) and 4-methylene-L-glutamine levels and significantly decreased tetradecanoic acid (fatty acid metabolism) and 2-(acetamidomethylene)succinate (vitamin B metabolism) levels. Individual FC, FDR, and VIP values for each significantly changed metabolite are displayed in Supplementary Table S1.

## 4. Discussion

By comparing nontargeted metabolomic profiles at fasting state and 2 h of OGTT, a considerably large number of distinct metabolites across different glycemic statuses were detected at 2 h of OGTT compared with the fasting state. Among the 544 detected metabolites, 80 changed significantly following OGTT (35 increased and 45 decreased), and the pattern of metabolite change diverged between T2D cases and noncases (normal glucose or prediabetes subjects).

To the best of our knowledge, this is the largest study investigating dynamic changes in the plasma metabolome during OGTT in a Chinese population. Most prior studies were conducted in Western populations and mainly focused on changes in metabolites in non-T2D subjects [12,13,14,18,29,30,31,32]. By contrast, the current study also analyzed changes of metabolites in individuals with newly diagnosed T2D, where less evidence had been available. In line with previous findings, we confirmed several metabolites that significantly responded during OGTT, including AMP, L-glutamate, and L-citrulline. Moreover, we identified a number of novel metabolites, including sphingosine, that were altered following glucose challenge. In addition, we revealed differences in dynamic changes of metabolites in response to OGTT across individuals of different glycemic statuses. Therefore, the present study provides novel insights regarding the different metabolic responses of subjects with different glucose hemostatic conditions, specifically in an Asian (including Chinese) population, which has different metabolic phenotypes and T2D susceptibility compared with Western populations.

The results showed that there were more different metabolites between T2D cases and those with normal glucose at 2 h of OGTT than at fasting state (22 metabolites vs. 1 metabolite). Levels of seven metabolites at 2 h of OGTT were also higher in T2D subjects than in prediabetes subjects. This phenomenon is likely to reflect the impairment of glucose homeostasis resilience of certain tissues/organs under glucose challenge, as indicated by the profoundly altered specific metabolite signals in T2D subjects. Among these different metabolites, glycerone was the only one positively associated with T2D at 0 h of OGTT, implying a potential role in generating advanced glycation end-products (AGEs) [33]. On the other hand, we found that five diabetes-related metabolites (amino acids: homocysteine [34] and glutamate [35]; TCA cycle metabolites: succinate [36] and malate [37]; and pyruvate [38]) previously reported at fasting state were significantly elevated in T2D cases at 2 h but not at 0 h of OGTT in the current study. This discrepancy might reflect different stages of impaired flexibility or resilience of the tissues or organs involved in regulating glucose homeostasis. It was unclear whether altered profiles of these metabolites and the related pathways would become evident only under a challenge such as OGTT in our newly diagnosed T2D cases. Moreover, the limited sample size of the current study might have been another reason. Nevertheless, we identified several novel metabolites, including erythrulose and linatine, that were significantly higher in individuals with T2D than in those with normal glucose. Although the underlying mechanisms were not fully elucidated, a study in a mouse model showed that levels of erythrulose, as a degradation product of ascorbic acid, increased during the pathogenesis of T2D [39]. In fact, increased degradation of ascorbic acid may contribute to accelerated oxidative damage [40]. Moreover, erythrulose was found to promote AGE formation via reaction with protein amino groups [41]. As an antagonist of vitamin B_6_, linatine at high circulating levels may downregulate vitamin B_6_ yield and thereby affect glucose-stimulated insulin secretion [42]. Further studies are needed to confirm our findings and to provide interpretations with respect to the mechanisms involved.

In addition to different profiles of metabolites across individuals with different glycemic statuses, we found that 80 metabolites significantly responded to glucose challenge. Among the 35 metabolites with significantly increased relative abundance, tauropine, derived from taurine, showed the most prominent elevation. Moreover, hypotaurine, the precursor of taurine, also showed significantly increased levels in T2D subjects at 2 h of OGTT in our study. Significant increases in taurine levels have been consistently observed after intake of glucose or sugar-sweetened beverages in previous studies [43,44]. Indeed, an in vivo study showed that taurine could improve glucose homeostasis by reducing the synthesis of AGEs or preventing mitochondrial dysfunction in β-cells [45]. Of note, the increase in levels of another metabolite, 5′-methylthioadenosine, could be explained by activation of adenosylmethionine synthase upon glucose intake and upregulated conversion of S-adenosylmethionine to 5′-methylthioadenosine [46]. Interestingly, we observed elevated levels of methane-metabolism-related metabolites, namely 5,10-methylenetetrahydromethanopterin and APMF-Glu, at 2 h of OGTT; this could be attributed to stimulation of methanogenesis by glucose loading. The increase in 3-phospho-D-glycerate, a glycolysis-related metabolite, may reflect activation of pathways during switching from the fasting to the re-feeding phase [12]. On the other hand, glucose challenge in the current study significantly reduced concentrations of 45 metabolites. Several of these have been previously reported, including metabolites involved in amino acid metabolism (glutamate and citrulline), purine metabolism (AMP and xanthosine), and fatty acid oxidation (tetradecanoic acid, hexadecanoic acid, (9Z)-hexadecenoic acid, erucic acid, octanoylcarnitine, and palmitoylcarnitine) [9,13,32]. The changes in these metabolites might be attributed to glucose-induced shifting from protein catabolism to protein anabolism, as well as inhibition of triglyceride catabolism and fatty acid β-oxidation. In addition to the previously reported metabolites, we also identified several novel ones, including sphingosine. As the precursor of sphingosine-1-phosphate (S1P), sphingosine plays an important part in sphingolipid metabolism [47]. In vitro studies revealed that elevated levels of sphingosine could inhibit glucose uptake in muscle cells, while S1P could upregulate glucose-stimulated insulin secretion in pancreatic β-cells [48,49]. Therefore, reduced levels of sphingosine might reflect elevated catalytic activity of sphingosine kinases following glucose challenge, which would promote transformation from sphingosine to S1P and consequently accelerate insulin secretion. Overall, these significantly glucose-responsible metabolites might provide subtle insights into the process of regaining glucose homeostasis.

Notably, our study showed different patterns of significantly changed metabolites between T2D cases and noncases. For instance, 4-methylene-L-glutamine, which is mainly involved in the C5-branched dibasic acid metabolism pathway related to carbohydrate metabolism in microbiota, was significantly increased in T2D subjects only [50]. T2D subjects also showed a smaller magnitude of decline in AMP levels compared with normal glucose and prediabetes subjects. This difference may have been due to impaired insulin sensitivity in T2D cases, as insulin was previously shown to downregulate AMP in rat hepatocytes in an in vitro study [51]. Furthermore, insulin resistance might also explain the blunted decline in D-glycerate (pentose phosphate pathway) and alpha-D-galactosyl-(1->3)-1D-myo-inositol (galactose metabolism) levels in T2D subjects. In addition to the fact that T2D and non-T2D subjects might have differently regulated metabolic pathways—including those involving amino acids (4-imidazolone-5-acetate, L-citrulline, β-citryl-L-glutamate), fatty acids (tetradecanoic acid, L-palmitoylcarnitine), vitamin B (thiamin triphosphate, 2-(acetamidomethylene)succinate), and purine (5-ureido-4-imidazole carboxylate, hypoxanthine)—it remains to be elucidated whether or to what extent personal variations in the resilience of glucose homeostasis could influence these significantly changed metabolites.

Strengths of the current study included the fact that we characterized patterns and changes of a large number of metabolites before and after glucose challenge among subjects with different glycemic statuses (normal glucose, prediabetes, and newly diagnosed T2D subjects). Moreover, repeated metabolomic profiling at 0 and 2 h of OGTT allowed subjects to serve as their own controls and eliminated confounding factors. Our study also had limitations. First, owing to limited resources, we only collected blood samples at 0 and 2 h of OGTT for metabolomics analysis, and some important metabolic signals at other time points may have been missed. Second, the nontargeted method was unable to accurately quantify the absolute level of a given metabolome. Third, the changes observed could be explained only for a limited number of metabolites, and mechanistic links for most metabolites were unclear. Last, the current study population was recruited from Bijie, Guizhou, and the findings of our study may not be generalizable to other populations.

## 5. Conclusions

In summary, we found several differences in levels of metabolites between T2D and normal glucose or prediabetes subjects at 0 and 2 h of OGTT, as well as a panel of metabolites that significantly changed with different patterns for different glycemic statuses during OGTT. Levels of metabolites under glucose challenge might provide more information than those in fasting state alone.

## Figures and Tables

**Table 1:** Characteristics of participants according to glycemic statuses.

	Total (*n* = 581)	Normal Glucose (*n* = 234)	Prediabetes (*n* = 281)	T2D (*n* = 66)	*p*
Age, year	48.3 ± 10.1	47.2 ± 10.2	48.3 ± 10.2	52.2 ± 8.2	0.002 ^a,b^
Male, *n* (%)	454 (78.1)	175 (74.8)	231 (82.2)	48 (72.7)	0.067
Current smoker, *n* (%)	351 (60.4)	137 (58.5)	179 (63.7)	35 (53.0)	0.211
Current drinker, *n* (%)	303 (52.2)	110 (47.0)	162 (57.9)	31 (47.0)	0.033 ^c^
Years of education, *n* (%)					0.501
0–6 year	124 (21.3)	50 (21.4)	56 (19.9)	18 (27.3)	
7–9 year	175 (30.1)	72 (30.8)	81 (28.8)	22 (33.3)	
≥10 year	282 (48.5)	112 (47.9)	144 (51.2)	26 (39.4)	
BMI, kg/m^2^	24.5 ± 3.5	24.0 ± 3.6	24.7 ± 3.3	25.1 ± 3.4	0.017 ^a,c^
Family history of diabetes, *n* (%)	13 (2.2)	0 (0.0)	9 (3.2)	4 (6.1)	0.004 ^a,c^
Fasting glucose, mol/L	5.92 ± 1.12	5.15 ± 0.34	6.06 ± 0.43	8.15 ± 1.68	<0.001 ^a,b,c^
2 h glucose (OGTT), mol/L	5.82 ± 2.74	4.77 ± 1.14	5.64 ± 1.79	10.47 ± 4.86	<0.001 ^a,b,c^
Fasting insulin, pmol/L	43.9 ± 27.9	38.3 ± 25.0	44.4 ± 24.9	61.7 ± 40.2	<0.001 ^a,b,c^
2 h insulin (OGTT), pmol/L	144.2 ± 143.5	112.7 ± 97.8	151.5 ± 144.4	227.5 ± 223.3	<0.001 ^a,b,c^
HOMA-IR	0.86 ± 0.57	0.72 ± 0.47	0.87 ± 0.48	1.33 ± 0.94	<0.001 ^a,b,c^
HOMA-B	60.0 ± 27.9	69.1 ± 30.0	56.3 ± 23.5	43.8 ± 26.5	<0.001 ^a,b,c^

Data are presented as mean ± SD (standard deviation), *n* (%), or median (IQR). Percentages may not sum to 100 because of rounding. Proportions were compared by chi-squared test, means by ANOVA, and medians by Kruskal–Wallis test. (a) Significantly different between T2D and normal glucose subjects (*p* < 0.05); (b) significantly different between T2D and prediabetes subjects (*p* < 0.05); (c) significantly different between prediabetes and normal glucose subjects (*p* < 0.05).

**Table 2:** Significantly different metabolites among subjects with different glycemic statuses at 0 and 2 h of OGTT.

Comparison	OGTT Time Point	Metabolite	OR per SD (95% CI)	FDR	VIP
T2D vs. normal glucose	0 h	Glycerone	2.02 (1.45–2.87)	0.025	3.31
	2 h	Xanthosine 5′-phosphate	1.76 (1.27–2.50)	0.033	2.18
		5-(L-Alanin-3-yl)-2-hydroxy-cis,cis-muconate 6-semialdehyde	1.78 (1.25–2.59)	0.049	2.16
		Cellobionate	1.79 (1.26–2.60)	0.045	1.93
		Glycochenodeoxycholate 7-sulfate	1.79 (1.27–2.70)	0.049	1.92
		L-Homocysteine	1.79 (1.29–2.53)	0.024	2.15
		Linatine	1.81 (1.29–2.59)	0.027	1.93
		1-Hydroxy-2-methyl-2-butenyl 4-diphosphate	1.84 (1.28–2.68)	0.033	1.02
		(S)-Malate	1.86 (1.29–2.74)	0.033	2.35
		O-Carbamoyl-L-serine	1.88 (1.36–2.66)	0.012	2.68
		D-Erythrulose	1.90 (1.37–2.72)	0.012	1.82
		Sphingosine	1.99 (1.36–2.99)	0.024	1.91
		Benzoate	1.99 (1.36–3.04)	0.025	2.41
		Pyruvate	2.01 (1.37–3.03)	0.024	2.49
		Succinate	2.02 (1.40–3.02)	0.014	2.23
		D-Glucono-1,5-lactone	2.02 (1.42–2.96)	0.011	2.57
		Parapyruvate	2.03 (1.44–2.95)	0.007	2.53
		3-β-D-Galactosyl-sn-glycerol	2.19 (1.36–3.64)	0.046	2.17
		Propanoate	2.22 (1.58–3.21)	0.002	2.80
		4-Imidazolone-5-acetate	2.27 (1.57–3.38)	0.003	2.99
		Glycerone	2.32 (1.64–3.37)	0.001	3.35
		Porphobilinogen	2.40 (1.65–3.61)	0.002	2.42
		L-Glutamate	2.44 (1.69–3.66)	0.001	2.26
T2D vs. prediabetes	2 h	Glycochenodeoxycholate 7-sulfate	1.66 (1.27–2.21)	0.024	2.35
		Propanoate	1.77 (1.31–2.43)	0.024	2.39
		(S)-Malate	1.84 (1.34–2.60)	0.024	2.37
		Linatine	2.02 (1.40–2.99)	0.024	2.58
		Glycerone	2.06 (1.54–2.79)	8.79 × 10^−4^	3.43
		4-Imidazolone-5-acetate	2.09 (1.47–3.08)	0.016	2.72
		Porphobilinogen	2.24 (1.56–3.33)	0.008	2.72

ORs per SD and 95% CIs were calculated by logistic regression model with multivariate adjustment for age, sex, education, family history of diabetes, current smoking, current drinking, BMI, and batch. VIP was calculated by PLS-DA model. OGTT, oral glucose tolerance test; OR, odds ratio; SD, standard deviation; FDR, false discovery rate; VIP, variable importance in projection; T2D, type 2 diabetes.

## Data Availability

The data presented in this study are available on request from the corresponding author.

## Supplementary Materials

The following are available online at https://www.mdpi.com/article/10.3390/nu13051474/s1, Table S1: Metabolites significantly changed at 2 h of OGTT. Figure S1: Study flow diagram. Figure S2: Venn diagram of significantly increased metabolites in total, normal glucose, prediabetes, and T2D subjects. Figure S3: Venn diagram of significantly decreased metabolites in total, normal glucose, prediabetes, and T2D subjects.
