# Supplementary material for: Changes in Plasma Metabolome Profiles Following Oral Glucose Challenge among Adult Chinese
Source: Nutrients. 2021 Apr 27;13(5):1474. doi: 10.3390/nu13051474 (PMC8146292; doi:10.3390/nu13051474)
Supplement: Supplementary file 1 [file nutrients-13-01474-s001.zip › nutrients-1157076-supplementary.pdf]

Supplementary Table 1. Metabolites significantly changed at 2-h of OGTT.

Metabolite	Total			Normal glucose			Prediabetes			T2D		
	FC	FDR	VIP	FC	FDR	VIP	FC	FDR	VIP	FC	FDR	VIP
Tauropine	1.79	3.78×10^{-18}	1.96	1.65	4.13×10^{-04}	1.39	1.94	3.27×10^{-14}	2.26	1.67	0.069	1.76
5'-methylthioadenosine	1.68	1.39×10^{-29}	2.43	1.86	3.13×10^{-17}	2.99	1.55	4.69×10^{-11}	1.92	1.66	0.031	2.14
D-mannose 1-phosphate	1.40	1.82×10^{-24}	2.43	1.34	1.53×10^{-06}	1.76	1.44	1.51×10^{-15}	2.62	1.47	0.005	2.77
Norbelladine	1.36	2.37×10^{-14}	1.56	1.33	2.84×10^{-05}	1.34	1.40	6.60×10^{-08}	1.56	1.31	0.093	1.62
Se-adenosyl-L-selenohomocysteine	1.34	2.60×10^{-26}	2.49	1.41	6.70×10^{-14}	2.57	1.30	1.12×10^{-11}	2.27	1.22	0.097	1.77
Cocaine	1.32	3.45×10^{-16}	1.72	1.36	2.89×10^{-09}	1.79	1.32	1.63×10^{-06}	1.56	1.24	0.094	1.29
CDP-choline	1.31	1.31×10^{-36}	2.89	1.35	3.56×10^{-19}	2.93	1.30	5.24×10^{-18}	2.8	1.22	0.138	1.62
5-acetamidopentanoate	1.30	8.24×10^{-17}	1.16	1.30	6.49×10^{-07}	0.97	1.29	7.95×10^{-08}	1.12	1.31	0.03	1.49
Nicotinamide D-ribonucleotide	1.26	1.62×10^{-09}	1.20	1.37	4.67×10^{-07}	1.48	1.23	5.13×10^{-05}	1.18	1.02	0.895	0.18
Glutaral	1.25	2.63×10^{-17}	1.16	1.28	2.88×10^{-09}	1.24	1.22	6.95×10^{-07}	0.96	1.30	0.067	1.24
Se-methylselenomethionine	1.24	1.60×10^{-19}	1.93	1.23	1.94×10^{-08}	1.74	1.25	1.35×10^{-10}	1.96	1.26	0.168	1.50
Beta-alanyl-L-arginine	1.24	5.53×10^{-26}	2.18	1.28	1.68×10^{-13}	2.17	1.24	5.67×10^{-13}	2.10	1.16	0.183	1.40
Coumaryl acetate	1.24	2.24×10^{-21}	2.25	1.30	4.14×10^{-12}	2.50	1.22	1.01×10^{-09}	1.93	1.11	0.206	1.43
Gamma-glutamyl-beta-cyanoalanine	1.24	2.71×10^{-19}	2.11	1.17	4.04×10^{-04}	1.38	1.29	9.91×10^{-14}	2.45	1.25	0.035	2.12
3-amino-L-alanine	1.23	1.47×10^{-11}	1.65	1.28	2.44×10^{-05}	1.63	1.21	5.07×10^{-06}	1.55	1.14	0.282	1.30
3-phospho-D-glycerate	1.22	6.74×10^{-16}	1.87	1.19	2.67×10^{-05}	1.54	1.25	3.43×10^{-09}	2.01	1.19	0.064	1.60
Riboflavin	1.22	1.35×10^{-14}	1.56	1.20	7.83×10^{-06}	1.31	1.23	7.95×10^{-08}	1.67	1.21	0.112	1.20
(R,S)-nicotine	1.21	5.41×10^{-05}	0.88	1.15	0.215	0.50	1.24	5.80×10^{-04}	1.00	1.32	0.238	1.34
Pseudouridine	1.21	8.59×10^{-52}	2.85	1.19	4.15×10^{-18}	2.46	1.22	3.99×10^{-30}	3.01	1.22	0.002	2.36
N-amidino-L-aspartate	1.21	2.29×10^{-19}	2.06	1.24	3.09×10^{-09}	2.04	1.21	3.09×10^{-12}	2.15	1.08	0.602	0.73
N6-hydroxy-L-lysine	1.21	1.77×10^{-12}	1.66	1.21	5.14×10^{-04}	1.32	1.25	6.88×10^{-11}	2.10	1.05	0.951	0.08
5,10-methylenetetrahydromethanopterin	1.20	8.80×10^{-05}	0.89	1.28	4.49×10^{-05}	1.51	1.16	0.053	0.73	1.11	0.827	0.27
(24R,24(1)R)-fucosterol epoxide	1.20	3.96×10^{-08}	1.29	1.18	0.026	0.85	1.23	4.05×10^{-06}	1.56	1.15	0.238	1.26

Carnosine	1.20	4.74×10^{-06}	1.02	1.18	0.004	0.97	1.23	0.001	1.02	1.13	0.62	0.63
5-ureido-4-imidazole carboxylate	1.19	1.93×10^{-07}	0.80	1.23	0.002	0.81	1.14	0.01	0.59	1.25	0.013	1.37
2-pyrone-4,6-dicarboxylate	1.18	1.21×10^{-11}	1.59	1.23	2.14×10^{-06}	1.63	1.16	8.08×10^{-06}	1.54	1.08	0.587	0.75
L-2-methyltryptophan	1.17	4.28×10^{-15}	1.45	1.21	1.31×10^{-09}	1.66	1.15	7.80×10^{-06}	1.23	1.11	0.191	1.06
APMF-Glu	1.16	1.84×10^{-17}	1.96	1.17	2.01×10^{-08}	1.91	1.15	8.99×10^{-07}	1.63	1.23	0.004	2.87
4-hydroxy-2-oxohexanoic acid	1.16	1.08×10^{-09}	1.40	1.17	3.43×10^{-05}	1.47	1.13	0.002	1.00	1.24	0.02	2.28
Taxifolin	1.13	1.48×10^{-05}	1.01	1.09	0.08	0.69	1.14	0.004	0.96	1.26	0.036	1.88
Hypotaurine	1.13	5.34×10^{-06}	1.19	1.11	0.032	0.89	1.13	0.006	1.06	1.25	0.036	2.22
Cytidine	1.13	5.23×10^{-05}	0.76	1.05	0.358	0.27	1.21	3.39×10^{-05}	1.12	1.08	0.528	0.68
Dhurrin	1.12	4.11×10^{-04}	0.88	1.12	0.219	0.56	1.11	0.02	0.87	1.21	0.044	1.73
4-imidazolone-5-acetate	1.09	1.31×10^{-04}	0.93	1.06	0.141	0.58	1.09	0.027	0.82	1.27	0.034	2.18
4-methylene-L-glutamine	1.02	0.57	0.16	0.98	0.638	0.19	1.00	0.843	0.09	1.31	0.036	2.08
Lipoyl-AMP	0.84	9.56×10^{-14}	1.56	0.88	1.87×10^{-04}	1.29	0.83	1.85×10^{-07}	1.46	0.75	0.03	2.30
Thiamin triphosphate	0.83	1.04×10^{-14}	1.81	0.83	1.20×10^{-05}	1.59	0.86	7.24×10^{-06}	1.54	0.77	0.004	2.77
L-citrulline	0.83	5.73×10^{-18}	1.69	0.86	2.92×10^{-06}	1.40	0.83	7.84×10^{-10}	1.68	0.78	0.025	1.94
beta-citryl-L-glutamate	0.83	4.09×10^{-14}	1.54	0.83	1.14×10^{-06}	1.47	0.84	1.39×10^{-05}	1.32	0.79	0.014	1.99
L-2-aminoadipate	0.83	2.87×10^{-19}	1.87	0.86	5.68×10^{-05}	1.37	0.80	7.73×10^{-14}	2.15	0.83	0.109	1.55
Tetradecanoic acid	0.83	1.55×10^{-30}	2.19	0.83	3.48×10^{-11}	1.87	0.83	1.29×10^{-16}	2.19	0.79	0.009	2.32
Sinapoyl-CoA	0.82	7.95×10^{-09}	1.09	0.79	3.48×10^{-06}	1.26	0.84	6.03×10^{-05}	1.06	0.88	0.778	0.44
3-ketolactose	0.82	6.91×10^{-10}	1.35	0.80	1.07×10^{-05}	1.55	0.81	9.02×10^{-04}	1.06	0.88	0.229	1.22
Hypoxanthine	0.81	4.72×10^{-07}	1.01	0.79	3.78×10^{-04}	1.12	0.85	0.016	0.76	0.76	0.069	1.25
L-tryptophanamide	0.81	1.86×10^{-09}	1.26	0.78	1.21×10^{-05}	1.52	0.82	8.87×10^{-05}	1.16	0.92	0.866	0.25
D-glycerate	0.81	6.19×10^{-10}	1.21	0.76	2.73×10^{-08}	1.81	0.82	7.29×10^{-04}	0.90	0.96	0.881	0.21
Galacturonic acid	0.81	4.55×10^{-20}	1.53	0.80	9.65×10^{-08}	1.34	0.80	6.71×10^{-13}	1.86	0.89	0.459	0.53
alpha-D-galactosyl-(1->3)-1D-myo-inositol	0.80	1.34×10^{-07}	1.29	0.74	7.65×10^{-07}	1.83	0.85	0.054	0.70	0.86	0.349	1.20
2-(acetamidomethylene)succinate	0.80	2.45×10^{-24}	2.38	0.81	7.71×10^{-08}	2.01	0.81	5.99×10^{-13}	2.35	0.78	0.002	2.63

L-palmitoylcarnitine	0.80	9.13×10^{-30}	2.17	0.79	2.29×10^{-16}	2.33	0.81	1.99×10^{-12}	1.90	0.85	0.067	1.67
Biotin sulfoxide	0.80	8.65×10^{-15}	1.53	0.78	1.80×10^{-08}	1.64	0.79	3.78×10^{-07}	1.46	0.93	0.71	0.53
D-lysopine	0.80	2.19×10^{-08}	1.36	0.81	0.002	1.15	0.80	2.15×10^{-04}	1.32	0.79	0.087	1.69
sn-glycero-3-phosphocholine	0.80	4.87×10^{-12}	1.51	0.75	6.54×10^{-08}	1.77	0.81	9.63×10^{-08}	1.65	0.95	0.947	0.10
Hexadecanoic acid	0.79	8.50×10^{-25}	2.26	0.78	1.03×10^{-14}	2.40	0.80	4.29×10^{-11}	2.05	0.81	0.099	1.72
Xanthosine	0.79	3.78×10^{-46}	2.88	0.78	5.45×10^{-18}	2.59	0.79	2.76×10^{-24}	2.95	0.82	0.004	2.22
sn-glycerol 3-phosphate	0.79	2.89×10^{-31}	2.41	0.78	1.03×10^{-14}	2.38	0.78	2.05×10^{-19}	2.61	0.87	0.584	0.67
Lithocholic acid	0.79	2.43×10^{-18}	1.66	0.82	2.63×10^{-06}	1.40	0.77	2.79×10^{-10}	1.77	0.76	0.044	1.37
Tyramine	0.78	1.01×10^{-06}	1.21	0.71	1.97×10^{-05}	1.55	0.87	0.199	0.56	0.72	0.02	2.07
1-O-[2-(acetylamino)-2-deoxy-alpha-D-glucopyranosyl]-D-myo-inositol	0.78	5.79×10^{-12}	1.68	0.76	8.11×10^{-07}	1.73	0.78	2.10×10^{-05}	1.52	0.82	0.288	1.28
4-glutathionyl cyclophosphamide	0.77	9.82×10^{-11}	1.37	0.77	5.28×10^{-05}	1.27	0.77	4.02×10^{-05}	1.26	0.75	0.112	1.53
L-octanoylcarnitine	0.77	1.44×10^{-18}	1.86	0.74	6.50×10^{-10}	1.95	0.78	3.72×10^{-07}	1.61	0.82	0.044	1.53
Sedoheptulose 7-phosphate	0.76	1.19×10^{-21}	2.23	0.76	2.89×10^{-09}	2.08	0.78	6.08×10^{-09}	1.94	0.72	0.002	3.00
2-(alpha-hydroxyethyl)thiamine diphosphate	0.75	4.90×10^{-24}	2.41	0.75	1.02×10^{-09}	2.19	0.74	3.67×10^{-13}	2.43	0.79	0.079	1.87
Choline phosphate	0.75	5.30×10^{-37}	2.49	0.75	5.18×10^{-15}	2.26	0.73	1.14×10^{-24}	2.75	0.81	0.233	1.28
5-hydroxy-L-tryptophan	0.74	6.77×10^{-20}	1.88	0.71	2.88×10^{-09}	1.88	0.77	1.59×10^{-08}	1.67	0.74	0.03	1.81
(9Z)-hexadecenoic acid	0.74	5.65×10^{-49}	2.93	0.73	2.14×10^{-22}	2.90	0.75	7.18×10^{-23}	2.66	0.75	0.004	2.72
S-succinylglutathione	0.74	1.34×10^{-31}	2.59	0.70	7.13×10^{-19}	2.82	0.76	1.87×10^{-13}	2.36	0.83	0.174	1.45
1-(5'-phosphoribosyl)-5-amino-4-imidazolecarboxamide	0.73	2.08×10^{-36}	2.84	0.74	1.03×10^{-14}	2.61	0.71	1.56×10^{-20}	2.87	0.81	0.067	2.02
Erucic acid	0.73	3.55×10^{-24}	1.73	0.72	2.89×10^{-09}	1.74	0.73	5.68×10^{-14}	1.60	0.77	0.036	1.35
Digalacturonate	0.73	4.96×10^{-27}	2.59	0.72	3.58×10^{-12}	2.51	0.73	2.04×10^{-12}	2.38	0.74	0.02	2.43
N4-(acetyl-beta-D-glucosaminyl)asparagine	0.73	1.13×10^{-25}	2.24	0.70	2.14×10^{-12}	2.30	0.72	1.16×10^{-14}	2.24	0.89	0.584	0.74
Sakuranetin	0.73	1.22×10^{-20}	1.99	0.69	6.44×10^{-10}	2.09	0.74	1.04×10^{-10}	1.91	0.86	0.371	0.92
4-hydroxy-2-quinolinecarboxylic acid	0.72	1.65×10^{-53}	2.92	0.74	5.09×10^{-20}	2.69	0.71	4.32×10^{-29}	3.06	0.74	0.004	1.99

N-acetyl-L-2-amino-6-oxopimelate	0.71	1.43×10^{-26}	2.60	0.73	9.29×10^{-09}	2.16	0.68	2.50×10^{-17}	2.83	0.79	0.107	1.74
L-mimosine	0.65	2.28×10^{-39}	2.71	0.65	1.03×10^{-14}	2.42	0.63	2.09×10^{-21}	2.79	0.76	0.006	1.90
16-hydroxypalmitate	0.65	3.65×10^{-68}	3.95	0.67	3.69×10^{-25}	3.60	0.62	1.08×10^{-37}	3.92	0.67	3.86×10^{-04}	3.47
Glycocholate	0.63	1.38×10^{-73}	3.43	0.65	5.81×10^{-28}	3.28	0.61	1.08×10^{-37}	3.28	0.68	1.32×10^{-05}	2.87
L-glutamate	0.56	1.62×10^{-69}	3.68	0.52	1.63×10^{-31}	3.86	0.56	6.16×10^{-40}	3.69	0.71	0.08	1.54
Sphingosine	0.53	2.15×10^{-59}	3.56	0.49	5.16×10^{-29}	3.60	0.54	6.80×10^{-27}	3.32	0.63	0.005	2.48
AMP	0.50	5.01×10^{-41}	3.22	0.48	1.74×10^{-20}	3.26	0.49	1.41×10^{-20}	3.15	0.62	0.152	1.58

FC, fold change; FDR, false discovery rate; VIP, variable importance in projection. FC of metabolites is shown as the geometric mean of the ratio of the metabolites in each sample at 2-h and 0-h. FDR was calculated by a linear mixed effects model, and VIP was extracted from the PLS-DA results.

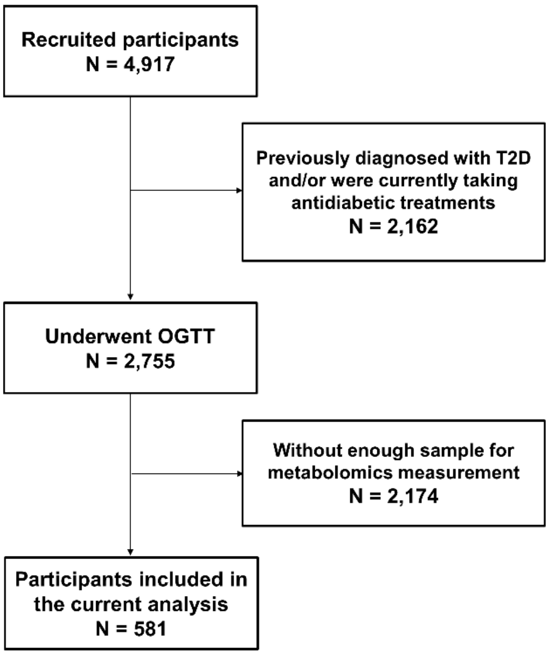

Supplementary Figure 1. Study flow diagram. We recruited 4,917 participants and 2,755 of them underwent OGTT. Finally, 581 participants with sufficient plasma sample were included in the current study.

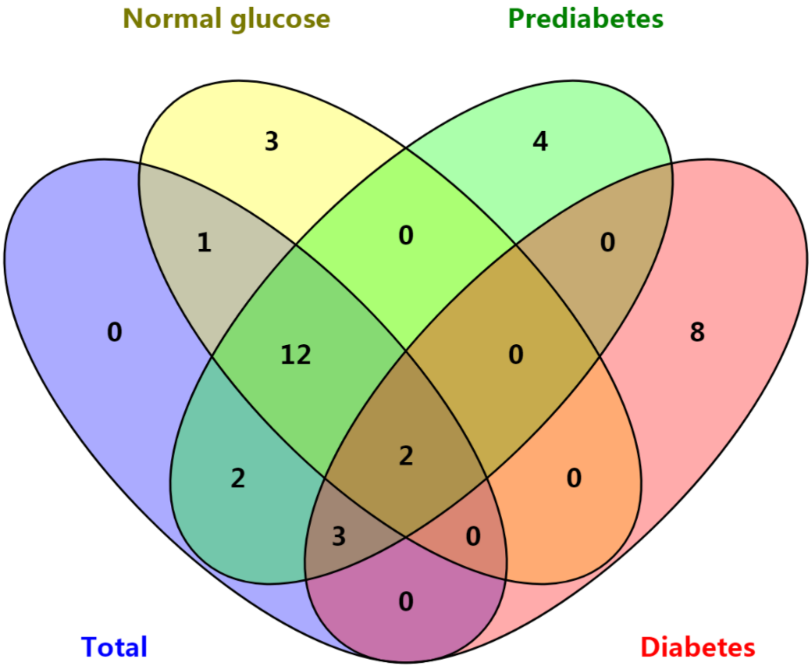

Supplementary Figure 2. Venn diagram of significantly increased metabolites in total, normal glucose, prediabetes, and T2D subjects.

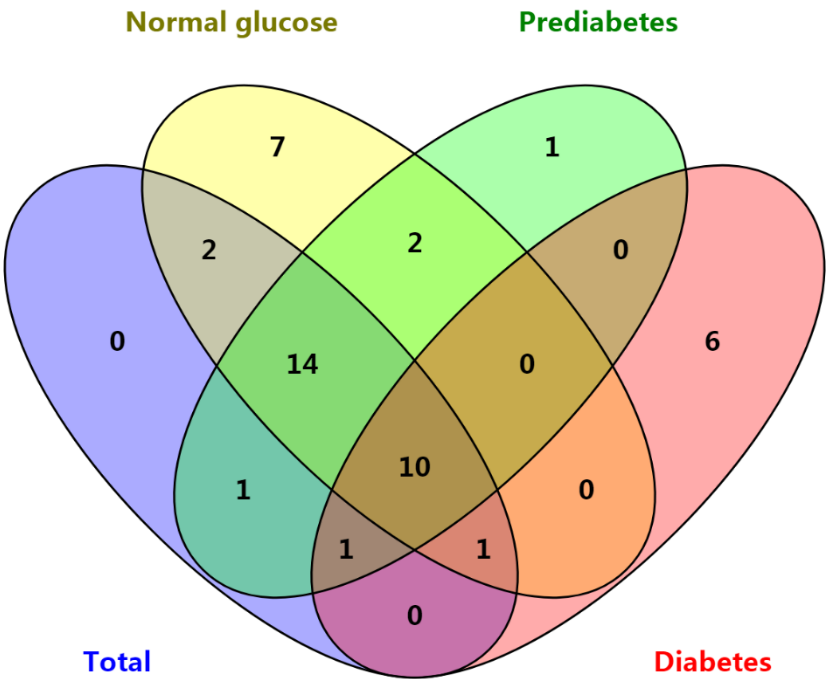

Supplemental Figure 3. Venn diagram of significantly decreased metabolites in total, normal glucose, prediabetes, and T2D subjects.
